# Association of Summer College Academic Enrichment Program Participation With Medical Student Diversity and Intent to Practice in Underserved Areas

**DOI:** 10.1001/jamanetworkopen.2020.34773

**Published:** 2021-02-05

**Authors:** Douglas Grbic, Norma Poll-Hunter, Natalie Felida, Dorothy A. Andriole

**Affiliations:** 1 Medical Education Research, Association of American Medical Colleges, Washington, DC; 2 Diversity Policy and Programs, Association of American Medical Colleges, Washington, DC; 3 Workforce Studies, Association of American Medical Colleges, Washington, DC

## Abstract

This cohort study examines the association of participation in a summer college academic enrichment program with diversity of students in medical school and intent to practice in underserved areas.

## Introduction

US medical schools seek to enroll diverse student populations to address national physician workforce needs. As a strategy to increase diversity, many organizations and institutions sponsor premedical summer college academic enrichment programs (SCAEPs) that may address Liaison Committee for Medical Education Standard 3.3, Diversity/Pipeline Programs and Partnerships.^1^ We examined national medical school matriculant cohorts to assess whether participation in an SCAEP is associated with increased student diversity and graduation of students with sustained intentions to practice in underserved areas.

## Methods

This cohort study included US medical school matriculants from 2013 to 2015 who graduated through 2019 and voluntarily completed the Association of American Medical Colleges (AAMC) Matriculating Student Questionnaire^2^ and Graduation Questionnaire.^3^ The AAMC Human Subjects Office exempted the study from institutional board review because it did not constitute human participant research and used deidentified data. This study followed the Strengthening the Reporting of Observational Studies in Epidemiology (STROBE) reporting guideline.

We identified SCAEP participants in the Robert Wood Johnson Foundation Summer Medical and Dental Education Program (SMDEP), which is a cost-free, 6-week national program to strengthen academic proficiency and career development of students underrepresented in health care professions^4,5^, by using program reports to the AAMC and other SCAEP participants (excluding those in SMDEP) by Matriculating Student Questionnaire self-report.^2^ We examined bivariate associations between SCAEP participation and sex, race/ethnicity, and first-generation college graduate (FGCG) identity and examined factors associated with sustained career intention (Matriculating Student Questionnaire and Graduation Questionnaire self-report) to practice in underserved areas by multilevel logistic regression. *P* values were 2-sided, and statistical significance was set at *P* < .05. Deidentified individual-level data linked via unique AAMC identifiers were analyzed using Stata, version 15 (StataCorp LLC).

## Results

The study sample included 29 456 graduates (all but 3 [missing data on sex] of 29 459 study-eligible graduates and 55% of all 53 079 US matriculants from 2013 to 2015 who graduated through 2019). A total of 15 050 (51%) were women, 4375 (15%) were graduates from racial/ethnic groups that are underrepresented in medicine, and 4255 (14%) were FGCGs (Table 1). Among 29 456 graduates, 360 (1.2%) were participants in SMDEP and 3776 (12.8%) were participants in other SCAEPs. Women, graduates from racial/ethnic groups that are underrepresented in medicine, and FGCGs were overrepresented among SCAEP participants.

**Table 1.** Study Sample by Summer College Academic Enrichment Program Participation

Characteristic	Total sample, No. (N = 29 456)	Individuals, No. (%)			*P* value^c^
		No SCAEP participation (n = 25 320)^a^	SMDEP participation (n = 360)^a^	Other SCAEP participation excluding SMDEP (n = 3776)^a^^,^^b^	
Sex^d^					
Men	14 406	12 457 (86.5)	125 (0.9)	1824 (12.7)	<.001
Women	15 050	12 863 (85.5)	235 (1.6)	1952 (13.0)	
Race/ethnicity^d^					
Black or African American only	1508	968 (64.2)	175 (11.6)	365 (24.2)	<.001
Hispanic, Latino, or of Spanish origin only	1610	1237 (76.8)	68 (4.2)	305 (18.9)	
Other URM^e^	1257	1049 (83.5)	26 (2.1)	182 (14.5)	
White and/or Asian	22 717	19 991 (88.0)	79 (0.3)	2647 (11.7)	
Other or unknown^f^	2364	2075 (87.8)	12 (0.5)	277 (11.7)	
First-generation college graduate^d^					
Yes	4255	3584 (84.2)	124 (2.9)	547 (12.9)	<.001
No	24 507	21 167 (86.4)	230 (0.9)	3110 (12.7)	
Unknown^f^	694	569 (82.0)	6 (0.9)	119 (17.1)	

Abbreviations: SCAEP, summer college academic enrichment program; SMDEP, Summer Medical and Dental Education Program; URM, race/ethnicity underrepresented in medicine, including those who identify as American Indian, Alaska Native, Native Hawaiian, other Pacific Islander, Black, African American, Hispanic, Latino or of Spanish origin alone or in combination with any other race/ethnicity.

^a^ Percentages within row; totals may not add to 100% owing to rounding.

^b^ Other SCAEP participants were those who were not identified as SMDEP participants by program report to the Association of American Medical Colleges but who did indicate on the Association of American Medical Colleges Matriculating Student Questionnaire that they had participated in an SCAEP.

^c^ Evaluated by χ^2^ tests of association.

^d^ Sex, race/ethnicity, and first-generation college graduate status information was provided by the students when applying to medical school through the Association of American Medical Colleges’ American Medical College Application System.

^e^ Graduates in the other URM category include those who identified as American Indian or Alaska Native only (n = 50) and as Native Hawaiian or Other Pacific Islander only (n = 24) as well as those who identified as Black or African American; Hispanic, Latino, or of Spanish origin; American Indian or Alaska Native; or Native Hawaiian or Other Pacific Islander in combination with any other race/ethnicity.

^f^ Unknown refers to missing data.

Table 2 shows results of multilevel logistic regression models identifying factors associated with sustained intention to practice in underserved areas. In the multivariable model, graduates who were women (vs men: adjusted odds ratio [AOR], 1.9; 95% CI, 1.8-2.0), FGCGs (AOR, 1.5; 95% CI, 1.4-1.7), Black or African American only (vs White and/or Asian: AOR, 3.3; 95% CI, 2.9-3.7), Hispanic, Latino, or of Spanish origin only (vs White and/or Asian: AOR. 2.5; 95% CI, 2.2-2.9), other race/ethnicity underrepresented in medicine (vs White and/or Asian AOR: 1.7; 95% CI, 1.5-2.0), and SMDEP participants (vs non-SCAEP participants: AOR, 1.9; 95% CI, 1.5-2.5) were more likely to report sustained career intentions to practice in underserved areas.

**Table 2.** Factors Associated With Sustained Intention to Practice in an Underserved Area^a^

Characteristic	Individuals with sustained intention to practice in an undeserved area, No./total No. (%)^b^	*P* value^c^	OR (95% CI)^d^	
			Unadjusted	Adjusted
Total	4530/29 456 (15)	NA	NA	NA
Sex^e^				
Men	1606/14 406 (11)	<.001	1 [Reference]	1 [Reference]
Women	2924/15 050 (19)		1.9 (1.8-2.0)	1.9 (1.8-2.0)
Race/ethnicity^e^				
White and/or Asian	2898/22 717 (13)	<.001	1 [Reference]	1 [Reference]
Black or African American only	598/1508 (40)		4.0 (3.6-4.6)	3.3 (2.9-3.7)
Hispanic, Latino, or of Spanish origin only	439/1610 (27)		2.8 (2.4-3.2)	2.5 (2.2-2.9)
Other URM^f^	270/1257 (21)		1.9 (1.6-2.2)	1.7 (1.5-2.0)
Other or unknown^g^	325/2364 (14)		1.1 (0.9-1.2)	1.1 (0.9-1.2)
First-generation college graduate^e^				
No	3415/24 507 (14)	<.001	1 [Reference]	1 [Reference]
Yes	997/4255 (23)		1.7 (1.6-1.9)	1.5 (1.4-1.7)
Unknown^g^	118/694 (17)		1.6 (1.2-2.0)	1.4 (1.1-1.8)
SCAEP participation				
None	3703/25 320 (15)	<.001	1 [Reference]	1 [Reference]
SMDEP	160/360 (44)		4.0 (3.2-5.0)	1.9 (1.5-2.5)
Other SCAEP excluding SMDEP	667/3776 (18)		1.3 (1.1-1.4)	1.1 (1.0-1.2)

Abbreviations: NA, not applicable; OR, odds ratio; SCAEP, summer college academic enrichment program; SMDEP, Summer Medical and Dental Education Program; URM, race/ethnicity underrepresented in medicine, including those who identify as American Indian, Alaska Native, Native Hawaiian, other Pacific Islander, Black, African American, Hispanic, Latino or of Spanish origin alone or in combination with any other race/ethnicity.

^a^ Graduates in the “sustained intention from matriculation through graduation to work in underserved areas” group were those who responded “yes” to the item “Do you plan to work primarily in an underserved area?” on both the Association of American Medical Colleges Matriculating Student Questionnaire and the Association of American Medical Colleges Graduation Questionnaire; this group of graduates was compared with the reference group of graduates, consisting of those who responded “no” or “undecided” to this item on at least 1 of the questionnaires.

^b^ Numbers and percentages shown are for rows.

^c^ Evaluated by χ^2^ tests of association.

^d^ Odds ratios and 95% CIs were estimated with multilevel logistic regression; US Liaison Committee for Medical Education-accredited medical schools (n = 142) were the random effect (intercept only).

^e^ Sex, race/ethnicity, and first-generation college graduate status information was provided by the students when applying to medical school through the Association of American Medical Colleges’ American Medical College Application System.

^f^ Graduates in the other URM category include those who identified as American Indian or Alaska Native only (n = 50) and as Native Hawaiian or Other Pacific Islander only (n = 24) as well as those who identified as Black or African American; Hispanic, Latino, or of Spanish origin; American Indian or Alaska Native; or Native Hawaiian or Other Pacific Islander in combination with any other race/ethnicity.

^g^ Unknown refers to missing data.

## Discussion

Previous studies^5^ of SCAEPs have shown an increase in the likelihood of application and matriculation to medical school associated with SCAEP attendance. We observed an association between SCAEPs and increased student diversity and that SMDEP participants were more likely to report sustained career intentions to practice in underserved areas. Because career intention to practice in underserved areas at graduation has established predictive validity,^6^ our findings may inform medical schools’ investments in programs to recruit diverse students whose career goals align with national needs to increase access to care.

Our study was limited by small numbers of American Indian or Alaska Native only and Native Hawaiian or other Pacific Islander only graduates, precluding their examination as discrete groups. In addition, we could not further disaggregate the other SCAEPs (a heterogenous group of programs regarding, for example, duration, cost, and curricular content), which limits generalizability of observations for this group. In the context of the current coronavirus disease 2019 (COVID-19) pandemic and recent activism in response to anti-Black racism, SCAEPs such as SMDEP (whose participants comprised 11.6% of all Black or African American only graduates in our national sample) may play a particular role in medical schools’ efforts to recruit and educate a diverse physician workforce that will meet national health care needs.

## References

[1] Liaison Committee for Medical Education, Association of American Medical Colleges and American Medical Association. Functions and structure of a medical school: standards for accreditation of medical education programs leading to the MD degree. March 2020 Accessed December 11, 2020. https://lcme.org/wp-content/uploads/filebase/standards/2021-22_Functions-and-Structure_2020-11-2.docx

[2] Association of American Medical Colleges Matriculating Student Questionnaire. 2020 Accessed July 15, 2020. https://www.aamc.org/data-reports/students-residents/report/matriculating-student-questionnaire-msq

[3] Association of American Medical Colleges Graduation Questionnaire (GQ). 2020 Accessed April 15, 2020. https://www.aamc.org/data-reports/students-residents/report/graduation-questionnaire-gq

[4] Robert Wood Johnson Foundation Brown MH. Summer medical and dental education program. program results report. October 7, 2013 Accessed December 11, 2020. https://www.rwjf.org/en/library/research/2011/08/summer-medical-and-dental-education-program.html

[5] Cantor JC, Bergeisen L, Baker LC Effect of an intensive educational program for minority college students and recent graduates on the probability of acceptance to medical school. JAMA. 1998;280(9):772-776. doi:10.1001/jama.280.9.772 9729987

[6] Ko M, Edelstein RA, Heslin KC, Impact of the University of California, Los Angeles/Charles R. Drew University Medical Education Program on medical students’ intentions to practice in underserved areas. Acad Med. 2005;80(9):803-808. doi:10.1097/00001888-200509000-00004 16123457

